# Prevalence of Virulence Factors and Drug Resistance in Clinical Isolates of Enterococci: A Study from North India

**DOI:** 10.1155/2015/692612

**Published:** 2015-08-23

**Authors:** Tuhina Banerjee, Shampa Anupurba

**Affiliations:** Department of Microbiology, Institute of Medical Sciences, Banaras Hindu University, Varanasi 221005, India

## Abstract

Along with emergence of multidrug resistance, presence of several virulence factors in enterococci is an emerging concept. This study was undertaken to determine the prevalence of various virulence factors phenotypically and genotypically in enterococci and study their association with multidrug resistance. A total of 310 enterococcal isolates were studied, comprising 155 *E. faecium* and 155 *E. faecalis*. Antimicrobial susceptibility testing was done by disc diffusion and agar dilution method. Hemolysin, gelatinase, biofilm production, and haemagglutination were detected phenotypically and presence of virulence genes, namely, *asa1, gelE, cylA, esp*, and *hyl*, was detected by multiplex PCR. Of the total, 47.41% isolates were high level gentamicin resistant (HLGRE) and 7.09% were vancomycin resistant (VRE). All the virulence traits studied were found in varying proportions, with majority in *E. faecalis* (*p* > 0.05). Strong biofilm producers possessed either *asa1* or *gelE* gene. *gelE* silent gene was detected in 41.37% (12/29). However, increase in resistance was associated with significant decrease in expression or acquisition of virulence genes. Further, acquisition of vancomycin resistance was the significant factor responsible for the loss of virulence traits. Though it is presumed that increased drug resistance correlates with increased virulence, acquisition of vancomycin resistance might be responsible for reduced expression of virulence traits to meet the “biological cost” relating to VRE.

## 1. Introduction

Enterococci exert dual functions both as commensals and as pathogens. When inside the body, they are well adapted to an ecological complex niche in the gut, genitourinary tract, and oral cavity which is enriched with low redox potential [1]. In order to exert their pathogenic effects, the primary steps involved are adherence to the specific host tissue, followed by invasion. These require several interactions with host defence mechanisms in an adverse environment, thus requiring expression of various enterococcal traits, ultimately contributing to virulence.

Virulence in enterococci has been said to evolve in a “mode and tempo” similar to evolution of pathogenic lineages of other organisms, where a subpopulation with enhanced or attenuated virulence traits capable of causing infections predominate and emerge [2]. Just as antimicrobial resistance has been best characterized in* E. faecium*, the characterization of virulence traits has been best done in* E. faecalis*. Recently prevalence of virulence traits has been studied in enterococcal isolates from various sources and notable presence has been found in river water, clinical samples, dental plaques, food products, and so forth [3]. With the emergence of virulence traits in enterococci, several interesting facts have come into the forefront. While on one hand it is usually presumed that increased virulence is associated with increased antimicrobial resistance, on the other hand, cost benefit analysis has revealed that antimicrobial resistance and virulence are two different aspects of bacterial cell fitness and therefore increased antimicrobial resistance might not always be associated with increased virulence [2]. Studies relating to both the assumptions have been reported, yet no conclusions on the exact association of antimicrobial resistance and virulence have been derived.

Virulence factors in enterococci isolated from clinical as well as environmental samples have been scarcely done in India, though the prevalence of enterococcal infections has been steadily increasing [4]. However, even the limited studies document the widespread distribution of such traits [5, 6]. This study was undertaken to determine the prevalence of various virulence determinants among the clinical isolates of enterococci and their drug resistance.

## 2. Materials and Methods

### 2.1. Collection and Identification of Study Isolates

The present study was conducted in the Department of Microbiology and the 1200-bed tertiary care university hospital of Institute of Medical Sciences, Varanasi, north India. The duration of this study was from September 2010 to March 2014. The study was approved by the Institute Ethical Committee. Relevant samples were collected from patients attending the different outpatient and inpatient services of the hospital mostly with clinical diagnosis of urinary tract infections, septicaemia, and pyogenic infections. Samples were directly inoculated on blood agar, MacConkey agar, and cysteine lactose electrolyte deficient (CLED) agar, as per the nature of the specimen. Colony morphology and culture characteristics were observed macroscopically. Identification of genus* Enterococcus* was done based on Gram staining, cultural characteristics, and physiological and biochemical tests, namely, bile esculin hydrolysis, PYR hydrolysis, and growth in 6.5% sodium chloride and at pH 9.6 [7]. Further speciation was done by standard set of biochemical tests including arginine dihydrolase test, mannitol, sorbitol, sorbose, arabinose, raffinose, lactose, sucrose (Sigma, USA), and pyruvate (Hi Media, India) fermentation tests, according to Facklam Collins classification [8].

### 2.2. Antimicrobial Susceptibility Testing

Antimicrobial susceptibility testing was done by modified Kirby Bauer disc diffusion method with the following discs nitrofurantoin (NIT, 300 *μ*g) for urinary isolates only and linezolid (LNZ, 30 *μ*g) (Hi Media, India). Breakpoint MIC was performed against ampicillin (AMP) and ciprofloxacin (CIP) in MHA by agar dilution method for all the clinical isolates [9]. Screening for vancomycin resistant enterococci (VRE) and high level gentamicin resistant enterococci (HLGRE) was done on brain heart infusion (BHI) agar containing 6 *μ*g/mL vancomycin and 500 *μ*g/mL gentamicin, respectively, by agar dilution method [9]. Multidrug resistance was defined as resistance to three or more different classes of antibiotics [10].

### 2.3. Phenotypic Detection of Virulence Factors

Phenotypic detection of virulence factors was done as described previously by previous publications [3, 11–13]. Briefly, for detection of hemolysin, isolates were streaked on BHI agar enriched with 5% human blood. Beta hemolysis surrounding bacterial colonies, seen after 24-hour incubation at 37°C, was considered indicative of hemolysin production. Gelatinase production was seen on Todd Hewitt agar incorporated with 3% gelatin. Following 48 hours of incubation, transparent halo around the colonies was seen in positive isolates after flooding the plate with Frazier solution (15% HgCl_2_ and 20% HCl). Haemagglutination test was done by taking a loopful of growth from BHI blood agar and mixing gently with 25 *μ*L of 3% human erythrocyte suspension in phosphate buffered saline (PBS, pH 7.4) on a 96-well microtitre plate (Tarsons, India). Haemagglutination was seen after rotating the plates for 5 minutes and then keeping the plates at room temperature for 30 minutes. All the isolates were tested for biofilm production by semiquantitative adherence assay. An overnight culture in BHI was further diluted with 2% glucose. Thereafter, 200 *μ*L of these cell suspensions was transferred to a microtiter plate and incubated aerobically at 37°C for 24 hours followed by washing and drying. Growth was fixed with 95% ethanol and stained with 1% crystal violet solution for 15 min. Optical density of each well was measured at 450 nm using an automated ELISA reader. Wells with sterile BHI alone were considered as negative control and* Staphylococcus epidermidis* ATCC 35984 as a positive control. Strong biofilm producers were considered in those with OD values > 0.5. Moderate biofilm production was considered in those with OD values > 0.2 but < 0.5.

### 2.4. Genotypic Detection of Virulence Genes

One hundred randomly selected isolates susceptible to high strength gentamicin (HSG) and vancomycin and 100 randomly selected HLGRE isolates and all the VRE isolates were further studied for the presence of virulence genes. Following genes encoding virulence factors were analyzed by multiplex PCR as per previous protocol without modification [14],* asa1* (aggregation substance),* gelE* (gelatinase),* cylA* (cytolysin),* esp* (enterococcal surface protein), and* hyl* (hyaluronidase). As detection of virulence genes was based on previously standardized method, no positive control was used, but each reaction was repeated three times. PCR amplicons from few isolates were randomly sequenced to validate the PCR. All reagents but no DNA template served as negative control in each set of reactions. Amplification of 16SrDNA (Forward-TTGGAGAGTTTGATCCTGGCC, Reverse-ACGTCATCCCACCTTCTC) was used as an internal control to avoid any false negative results. For PCR reactions, approximately 60 ng of DNA was used to avoid any false positive results.

### 2.5. Statistical Analysis

Species prevalence and association of biofilm production with virulence traits were analyzed and compared by Chi square test. Presence of virulence genes and drug resistance was compared by Kruskal Wallis test. Further to find the source of variation Mann-Whitney *U* test was applied. All statistical analysis was done by SPSS version 15, SPSS Inc.

## 3. Results

A total of 313 enterococcal isolates were collected from urine (298), pus (10), and blood (5). Further study was conducted with the major species only. Of the total isolates, ampicillin resistance was more common in* E. faecium* showing 58.7% (91 of 155) resistance against 38.4% (58 of 155) in* E. faecalis* while nearly 58.06% (90 of 155) of the* E. faecium* and 65.8% (102 of 155)* E. faecalis* isolates were resistant to ciprofloxacin. Further, 83 (53.54%)* E. faecalis* and 64 (41.29%)* E. faecium* were HLGRE and 9 (5.8%)* E. faecalis* and 13 (8.3%)* E. faecium* were VRE as detected by agar screening method. None of the isolates were resistant to linezolid.

**Figure 1:**
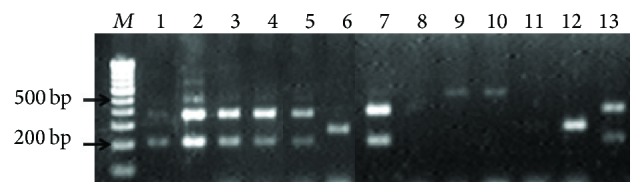
Gel electrophoresis showing virulence genes in the enterococcal isolates detected by multiplex PCR. Lane M: 100 bp ladder; Lane 1:* gelE* (213 bp) and* asa1* (376 bp) genes in* E. faecalis*; Lane 2:* gelE* (213 bp),* asa1* (376 bp),* esp* (510 bp), and* cylA* (688 bp) genes in* E. faecalis*; Lanes 3, 4, and 5:* gelE* (213 bp) and* asa1* (376 bp) genes in* E. faecium*; Lane 6:* hyl* (276 bp) gene in* E. faecalis*; Lane 7:* gelE* (213 bp) and* asa1* (376 bp) genes in MDR* E. faecalis*; Lane 8: absence of virulence genes in VRE* E. faecalis*; Lane 9, 10:* esp* (510 bp) gene in VRE* E. faecium;* Lane 11: absence of virulence genes in VRE* E. faecium*; Lane 12:* hyl* (276 bp) gene in VRE* E. faecium*; Lane 13:* gelE* (213 bp) and* asa1* (376 bp) genes in HLGR* E. faecalis*.

All the virulence traits under consideration were found both phenotypically and genotypically in varying proportions in the isolates as shown in Tables 1 and 2 and Figure 1. Phenotypic expression of virulence traits and majority of the virulence genes were found in* E. faecalis*, though there was no significant association between virulence factor and species of* Enterococcus* studied (*p* > 0.05). Amongst the virulence genes, as shown in Table 2,* asa1* and* gelE* were the most prevalent ones in the susceptible isolates whereas* gelE* and* hyl* were more frequently seen in the resistant isolates. However, none of the isolates harboured all the virulent genes and 44 isolates had no virulent genes, though phenotypically they demonstrated hemolysis (22 isolates), haemagglutination (19 isolates), and biofilm production (15 isolates). Ten enterococcal isolates did not show any virulence factors both phenotypically and genotypically.

Association of virulence genes with strong biofilm production (OD > 0.5) was studied as shown in Table 3. Majority of the strong biofilm producers possessed either* asa1* or* gelE* gene. Multiple virulence genes were not seen in association with biofilm production. Moderate biofilm producers (OD > 0.2 < 0.5) were not associated with virulence genes.

On analysis of resistance profile of the isolates with virulence traits, decreasing expression and possession of virulence genes was seen with increasing multidrug resistance (MDR) (Table 4). Isolates with resistance to ampicillin, ciprofloxacin, and HSG but susceptible to vancomycin and nitrofurantoin expressed more virulence factors than vancomycin resistant ones. This difference in the resistance profile and virulence genes carried by the isolates were significant for both the species (*p* < 0.05). Further, Mann-Whitney *U* component showed that acquisition of vancomycin resistance along with MDR phenotype (AMP^R^  CIP^R^  HSG^R^  NIT^R^) is the source of variation.

## 4. Discussion

Enterococcal infections are one of the most important global health problems causing considerable morbidity in the general population. We studied the two major species associated with most of the enterococcal infections, namely,* E. faecalis* and* E. faecium*. Whereas* E. faecalis* is the commonly reported species,* E. faecium* is equally gaining importance and is the major isolated species in many centres, as in ours [15] and in this study. From epidemiological point of view, increasing drug resistance in enterococci has been held responsible for the emergence of* E. faecium* as a dominant species especially as VRE isolates. Along with this, virulence traits are less prominent in* E. faecium* than* E. faecalis* [3]. However, even though virulence traits were more frequently present in* E. faecalis* in our study, there was no significant association relation between virulence factors and enterococcal species studied (*p* > 0.05).

Bacterial adherence to host tissues is a very vital step in initiation of any infection process. In this respect, enterococci have several options. Most of the virulence genes commonly harboured, namely,* esp*,* cylA*, and* asa1,* have been associated with adherence. Often these genes are located on specified region of the genomes, distinctively marked as “pathogenicity island” [16].* Esp* helps in adherence to the bladder wall via mucin and uroplakin receptors thus helping enterococcal colonization and persistence in urinary tract [17]. Similarly,* asa1* has been seen to help in adherence to renal cells [16]. Though cytolysin expression is concurrent with hemolysin production, different components of the cytolysin operon containing five genes (*cyl1*,* cyl2*,* cylA*,* cylM,* and* cylB*) have been attributed to this hemolysis [18], not necessarily a single one. This was reflected in our study where* cylA* gene was only present in 5% of isolates whereas hemolysin production was seen in 39% of the isolates. In another study, almost 75 isolates with hemolysin activity were negative for* cylA* gene suggesting possible role of other genes in hemolysin activity [19]. Additionally this finding emphasizes the need for testing virulence factors both phenotypically and genotypically.

Various studies on virulence factors of enterococci have currently reported their widespread distribution. As compared to our study, in a recent study from south India, hemolysin production was seen in 82% of the clinical isolates, while gelatinase production was demonstrated in 40.6% of the isolates [5]. Even commensal isolates of enterococci showed 44% hemolysin production and 32% gelatinase production in another study [20].

Besides the virulence genes, RBC agglutination property is a predictable measure of adherence [12]. Haemagglutination was seen in 21.93% of the isolates. The mannose and glucose receptors in the urinary tract help in adhesion of the enterococcal isolates showing haemagglutination activity. It is of interest to know that these adhesins are often transferable in the form of plasmids to other strains [21]. Property of haemagglutination as a virulence factor of enterococci has been studied less frequently, and total absence of this factor has been reported in one of the above-mentioned studies on endodontic isolates [22].

Biofilm formation in enterococci is one of the several defense mechanisms to evade action of antibiotics and help in persistence of infections, especially on indwelling catheters [23, 24]. Interestingly, majority of the strong biofilm producing enterococci were isolated from patients with UTI with indwelling catheters (29 of the 62 biofilm producers, 46.7%), of which 22 (75.86%) were indoor patients. Indwelling urinary catheters, use of intravascular devices, and prolonged hospitalization have been studied to be significant risk factors for enterococcal infections [25]. Even higher rates of biofilm formation (86.6%) have been reported from India among the urinary isolates of enterococci [16], whereas another study has reported biofilm formation by all the endodontic enterococcal isolates [22].

Though all the genetic determinants, namely,* gelE*,* esp,* and* asa1,* have been associated with biofilm formation in enterococci, in this study* gelE* singly or in combination was the most prominent virulence determinant amongst the biofilm producers. While all the gelatinase producers were harbouring the* gelE* gene, the reverse was not true. The detection of* gelE* silent gene in 41.37% (12/29) of the isolates could be accounted for by several reasons as determined by other studies [26], which clearly show that expression of* gelE* is triggered in late exponential phase at high cell densities. Their presence in the clinical isolates is equally important as their expression under optimum conditions* in vivo* might increase severity of infections. In contrast to studies demonstrating* esp* dependent biofilm formation [24], we found* esp* independent biofilm formation in most of the isolates.

Owing to changing epidemiology, increasing drug resistance in enterococci has been held responsible for the emergence of* E. faecium* as a dominant species especially as VRE isolates. One such fact is the emergence and rapid increase in VRE from clinical infections to the extent that greater than 25% of enterococcal infections are due to VRE* E. faecium* in ICU of US [27]. Along with this, virulence traits have been observed to be more prominent in* E. faecium* than* E. faecalis* [3]. Virulence gene “*esp*” which is considered as a marker for an epidemic clone of* E. faecium* that has spread across the countries [28] was not largely detected in this study. This might be due to early introduction of this clonal complex in our setup.

A study on environmental enterococcal isolates from Ganges water in and around Varanasi, India, our study place, showed the prevalence of multiple enterococcal species and multiple virulence traits of different* Enterococcus* spp. obtained from surface water [29]. The same study also showed increased prevalence of VRE in river water in sites where there were agriculture farms, intensive livestock, and swine farming in the locality and hospital sewage discharge points. Widespread dissemination of virulence markers in river water might have been due to natural horizontal transfer of virulence traits from pathogenic enterococci from hospitals thus leading to evolution of MDR and multivirulent enterococci [29].

Antibiotic resistance determinants, cytolysin toxin production, gelatinase production, aggregation substance, and enterococcal surface protein are some of the traits that have infiltrated into the species to varying extent, thus increasing pathogenicity of enterococci. As majority of the virulent genes are plasmid-borne, their infrequent possession amongst the unusual enterococcal species probably indicates that the emerging species have not yet faced the widespread dissemination of virulent determinants like the major species. Another explanation of this aspect could be the cost of fitness of these emerging organisms, while emerging drug resistance in these isolates is sufficient enough for better survival, eliminating the requirement of additional virulence traits.

**Figure 2:**
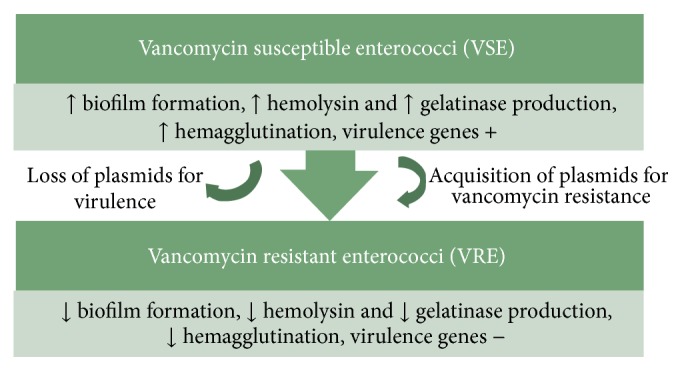
Probable mechanism of regulation of drug resistance and virulence in enterococci.

As has been suggested previously, the usual presumption is that increased antimicrobial resistance correlates with increased virulence along with increased mortality. But because studies have shown that mortality is independent of resistance profile especially in cases of VSE and VRE and MSSA and MRSA, it was speculated that increase in one aspect of survival fitness reduces the other [3]. In this context, a very relevant finding should be mentioned. It has been seen that, in case of community acquired MRSA (CA-MRSA), the genetic region associated with beta lactam resistance, namely, “SCCMecA,” is smaller in size and more transferrable as compared to hospital acquired MRSA (HA-MRSA). Consequently, in order to meet the “cost of fitness” for carrying a larger element, HA-MRSA are low toxin producers or possessors of other virulence elements as compared to CA-MRSA [30]. Similarly, it has been seen for enterococci that strict regulation of expression of resistant determinants considerably lowers the “biological cost” relating to VRE. Such type of strict regulation is more common in horizontally transferred elements in enterococci like drug resistance and virulence factors [31]. Therefore, increase in resistance profile could be associated with decrease in virulence as shown in Figure 2. This could be explained by the fact that expression of vancomycin resistance is biologically costly for enterococci and so this mechanism is tightly regulated and acquired only when it is essential for bacterial survival [31]. To further reduce the fitness cost, gain of these plasmids is compensated by loss of virulence plasmids.

Though not clearly established, the above-mentioned fact can be justified from similar findings in other studies from India and elsewhere. In a study with the aim to determine the difference in virulence factors expressed by VRE and VSE, it was clearly found that factors like hemolysin production and biofilm formation were more in VSE than VRE isolates, though the difference was not statistically significant [6]. Similarly, in another study, it was observed that presence of* hyl* gene, along with simultaneous presence of* hyl* and* esp* genes and bacterial adhesion to Vero cells, was more in VSE than VRE isolates, both from clinical and environmental sources [32]. In a pilot study on enterococcal UTI from the same centre, VRE isolates possessed significantly fewer virulence factors than the susceptible isolates [33].

It should be noted that majority of the drug resistant determinants in enterococci and virulence genes are plasmid-borne with immense ability for genetic exchange both intragenically and intergenically [1]. Consequently acquisition of one set of plasmid may lead to loss of the other either due to incompatibility or due to fitness cost benefits. These speculations can only hold true when further research is done on these aspects of virulence and enterococci. Much remains to be revealed about survival and complex interplay between drug resistance and virulence factors.

## 5. Conclusions

From this study, we concluded that virulence determinants have been widely prevalent in enterococcal isolates from clinical origin. However, there appears to exist a strict and complex regulation mechanism of expression of these virulence determinants especially with respect to antibiotic resistance in enterococci so that maximum benefit is obtained at minimum cost while exerting their pathogenic effects on host cells. This study summarizes the observational association of virulence and drug resistance in enterococci and emphasizes the need for further research on the role of horizontal gene transfer and defence mechanisms of enterococci.

## Figures and Tables

**Table 1:** Phenotypic detection of virulence factors in enterococcal isolates from clinical samples.

Species	Hemolysin production (%)	Gelatinase production (%)	Hemagglutination (%)	Biofilm production (%)
*E. faecium *(155)	36 (23.22)	13 (8.3)	35 (22.58)	39 (25.16)
*E. faecalis *(155)	62 (40)	15 (9.6)	33 (21.29)	42 (27.09)
Total (310)	**98 (31.61)**	**28 (9.03)**	**68 (21.93)**	**81 (26.12)**

**Table 2:** Genotypic detection of virulence determinants in clinical isolates of enterococci.

Antibiotic susceptibility	Species	*asa1*	*gelE*	*cylA*	*esp*	*hyl*
*Sensitive to vancomycin and HSG*	*E. faecalis (46)*	17 (36.95%)	14 (30.43%)	3 (6.52%)	8 (17.39%)	7 (15.21%)
	*E. faecium (54)*	12 (22.22%)	15 (27.77%)	2 (3.7%)	6 (11.11%)	7 (12.96%)

*HLGRE*	*E. faecalis (45)*	9 (20%)	14 (31.11%)	1 (2.2%)	3 (6.6%)	16 (35.5%)
	*E. faecium (55)*	10 (18.18%)	19 (34.54%)	1 (1.8%)	8 (14.54%)	11 (20%)

**Table 3:** Association of biofilm formation with virulence genes.

Biofilm formation	Genotype profile	*E. faecalis* (26)	*E. faecium* (28)
+++	*gelE* ^+^ only	7 (26.92)	5 (17.85)
+++	*esp* ^+^ only	2 (7.69)	5 (17.85)
+++	*asa1* ^+^ only	8 (30.76)	6 (21.42)
+++	*asa1* ^*+*^ *gelE* ^*+*^	5 (19.23)	1 (3.5)
+++	*gelE* ^+^ *esp* ^+^	0	1 (3.5)
+++	*gelE* ^+^ e*sp* ^+^ *asa1* ^*+*^	0	1 (3.5)
++	*gelE* ^−^ *esp* ^−^ *asa1* ^−^	6 (23.07)	9 (32.14)

**Table 4:** Relation of antimicrobial resistance with virulence in enterococci.

Virulence factors	^a^AMP^R^ CIP^R^ HSG^R^		^a^AMP^R^ CIP^R^ HSG^R^ NIT^R^		^b^AMP^R^ CIP^R^ HSG^R^ NIT^R^ VAN^R^	
	*E. faecalis *(11)	*E. faecium *(10)	*E. faecalis *(6)	*E. faecium *(9)	*E. faecalis *(4)	*E. faecium *(4)
Hemolysin production	5	2	1	1	0	0
Gelatinase production	1	0	0	0	0	0
Hemagglutination	5	4	0	2	0	1
Biofilm production	3	4	1	3	1	0
*asa1*	3	5	1	2	0	1
*gelE*	2	4	1	2	0	0
*esp*	2	2	1	2	0	2
*cylA*	0	2	0	0	0	0
*hyl*	1	1	0	2	0	1

^a,b^Mean with the same letter is not significant for the entire column for both the species. Kruskal Wallis applied.
